# Comparative Effects between Oral Lactoferrin and Ferrous Sulfate Supplementation on Iron-Deficiency Anemia: A Comprehensive Review and Meta-Analysis of Clinical Trials

**DOI:** 10.3390/nu14030543

**Published:** 2022-01-27

**Authors:** Xiya Zhao, Xu Zhang, Teng Xu, Junjie Luo, Yongting Luo, Peng An

**Affiliations:** Key Laboratory of Precision Nutrition and Food Quality, Department of Nutrition and Health, China Agricultural University, Beijing 100193, China; 13051239388@163.com (X.Z.); zhangx94@cau.edu.cn (X.Z.); 13031129533@163.com (T.X.)

**Keywords:** lactoferrin, ferrous sulfate, iron

## Abstract

Ferrous sulfate is a commonly used iron supplement for the correction of iron-deficiency anemia but with frequent gastrointestinal side effects. Milk-derived iron-binding glycoprotein lactoferrin possesses well gastrointestinal tolerance and fewer side effects caused by the intake of high-dose iron. However, the underlying mechanism of the iron-enhancing effect of lactoferrin remains unclear. In addition, the comparative efficacies between lactoferrin and ferrous sulfate are also remained to be determined. We conducted a systematic review and meta-analysis on published intervention studies to investigate how lactoferrin modulate iron metabolism and evaluate the comparative effects between lactoferrin and ferrous sulfate supplementation on iron absorption, iron storage, erythropoiesis and inflammation. Lactoferrin supplementation had better effects on serum iron (WMD: 41.44 ug/dL; *p* < 0.00001), ferritin (WMD: 13.60 ng/mL; *p* = 0.003) and hemoglobin concentration (11.80 g/dL; *p* < 0.00001), but a reducing effect on fractional iron absorption (WMD: −2.08%; *p* = 0.02) and IL-6 levels (WMD: −45.59 pg/mL; *p* < 0.00001) compared with ferrous sulfate. In conclusion, this study supports lactoferrin as a superior supplement to ferrous sulfate regarding the improvement in serum iron parameters and hemoglobin levels. Considering the weak influence of lactoferrin on iron absorption, the anti-inflammation effect of lactoferrin may be the potential mechanism to explain its efficacy on iron status and erythropoiesis.

## 1. Introduction

Iron is a necessary trace element for all mammals and involved in many essential metabolic processes such as oxygen transport, mitochondrial respiration and enzymatic activities [1]. Therefore, the deficiency of iron will lead to the metabolic abnormality in all parts of human bodies. The most common manifestations of iron deficiency are fatigue, headache and paleness [2]. Iron deficiency (ID) impairs cardiac function in the elderly and neurocognitive development in infants [1]. Children suffering from severe iron deficiency are associated with cognitive dysplasia, delayed body development, and low productivity [3,4]. The depletion of iron will also restrict hemoglobin synthesis and result in iron-deficiency anemia (IDA) [5,6]. IDA is the unique preventable and theorical easier treatable of the main and more frequent years lived with disabilities (YLDs) in the whole world [7].

Anemia has already become a global public health concern [8]. One in third people worldwide are affected by anemia; pregnant women and children are the most susceptible populations due to the increasing demand of iron for fetus or body development [9]. About half of pregnant women are diagnosed with anemia in low- and middle-income countries [10], and it is estimated that global anemia prevalence is more than 40% in pre-school children [8,11]. Anemia is also the first and main preventable cause of maternal mortality. An analysis of 312,281 pregnancies in 29 countries shows that the odds of maternal death were twice as high in those women with severe anemia compared with those not [12]. According to World Health Organization, 50% of anemia worldwide is caused by iron deficiency. The lack of iron additionally raises the specific risks for the mother and the fetus during pregnancy, such as intra-uterine growth retardation, prematurity, fetoplacental miss ratio and peripartum blood transfusion [13]. Therefore, the reduction of anemia is one of the World Health Assembly Global Nutrition Targets for 2025 and of the Sustainable Development Goals, with the attention to iron supplement and other nutritional needs of adolescent girls, pregnant women and children. However, the global progress in reducing anemia is not on track for reaching the 2025 target [8,14].

Generally, there are 6 “right treatment” guidelines regarding iron supplementation: at the right moment (not only when anemia is present), at the right dose (ensuring efficacy and tolerance of different populations); the right moment (breakfast is not the best meal to intake oral iron considering gastrointestinal reaction); the right route (oral iron is not always the first option, dietetical supplements, fortified powders, delay clamping umbilical cord or intravenous iron administration have respective advantages [4]); the right pharmacology (not all the oral iron are equal, neither the intravenous iron); and the right management (identifying the causes of IDA and the health problem that may weaken the effect of iron supplementation, including obesity/overweight, inflammation, and infection) [15].

There are several approaches for iron supplementation, including dietary or intravenous iron administration. Considering cost-effectiveness and adherence, oral iron ingestion is the main therapy for pregnant women and children with ID or IDA. Oral iron therapies include different iron supplements such as ferrous sulfate, ferrous gluconate and some other iron abundant substances. However, the absorption of iron across intestine epithelium is restricted by ferrous iron ion channel and influenced by body iron needs. Inorganic, non-heme iron from food is absorbed by duodenal enterocytes through divalent metal transporter 1 (DMT1), exported by ferroportin (FPN) and bound by transferrin within blood circulation [16]. The major delivery target of transferrin-bound iron is erythroid in the bone marrow, which can acquire transferrin through transferrin receptor 1 (TfR1) on the erythroid cell surface. Senescent erythrocytes will be lysed by macrophages and iron can be recycled into circulation. Excess iron is stored in the liver as ferritin. High level of iron storage will trigger hepcidin gene (*HAMP*) transcription to block iron absorption from intestine and iron recycling from macrophages [17]. Hepcidin can bind to FPN, trigger the internalization and degradation of FPN, reducing circulating iron level [18,19]. In addition, hepcidin plays an important role in innate immunity. The expression of hepcidin can be induced by inflammatory cytokines during infection, such as interleukin-6 (IL-6) [20,21], which is regarded to deprive invading microorganisms of iron. During nutrient scarcity, the microbiota can also compete with their hosts for iron by inhibiting host iron transport and storage [22].

Although the regulatory mechanism of iron absorption, utilization, storage and regulation has been discovered, ferrous sulfate is still a very frequently used oral iron supplement for the correction of ID or IDA, sometimes cooperated with vitamin C as adjuvant therapy. However, ferrous iron supplements frequently lead to serious gastrointestinal side effects such as vomiting, nausea, epigastric discomfort and further results in low patients’ compliance [23]. In recent years, milk-derived iron-binding glycoprotein lactoferrin has been used as iron supplement for the correction of ID/IDA [24,25,26]. Lactoferrin shares structural similarities with transferrin [27]. Lactoferrin displays high iron-binding affinity within a wide range of pH values, and the iron-binding capacity can be maintained after heating [1]. In commercially available lactoferrin, lactoferrin attains 10–20% iron saturation. Due to the iron-binding properties, it proposed that lactoferrin can enhance intestinal iron absorption and improve hemoglobin production [1]. However, the underlying mechanism of the iron-enhancing effect of lactoferrin remains unclear. Lactoferrin is a relatively large protein (~80 kD) that is unlikely to cross blood/gut barrier and directly contribute to iron supplementation [28]. Regarding the comparative efficacy between oral lactoferrin and traditional ferrous sulfate therapy on iron absorption, iron storage, erythropoiesis and inflammation, a high-quality comprehensive assessment is still needed to elucidate the potential mechanism of iron-enhancing effect of lactoferrin. Therefore, we conducted a systematic review and meta-analysis on published intervention studies to determine the comparative effects between lactoferrin and ferrous sulfate supplementation in the treatment of ID and IDA.

## 2. Materials and Methods

This systematic review was conducted in accordance with the Preferred Reporting Items for Systematic Reviews and Meta-Analyses (PRISMA) statement. Following keywords were used to perform a systematic search in PubMed, Web of Science and Cochrane Library: lactoferrin AND (iron OR anemia OR hemoglobin OR ferritin). Only clinical trials written in English were included and searching result was updated monthly until submission for publication. Literature search and evaluation were performed by two reviewers independently according to title, abstract and full text. The third reviewer re-examined searching result. Following criteria were used in literature evaluation: (1) clinical trials conducted in ID/IDA or healthy individuals; (2) study reporting baseline and post-intervention results on iron status (including at least one of the following outcomes: hemoglobin concentration; total serum iron (SI); serum ferritin (SF); and fractional iron absorption (FIA); (3) the intervention substance was lactoferrin or bovine lactoferrin, and ferrous sulfate must be used in control groups. The following exclusion criteria were used: (1) no control group in study; (2) animal research; (3) the mean change and standard deviation (SD) in the outcomes is unreported or cannot be calculated.

### 2.1. Data Extraction

Two independent reviewers screened and evaluated the full text of eligible research, and evaluation results were re-examined by another 2 reviewers. The primary outcomes included SI, SF concentration and hemoglobin concentration. Secondary outcomes included interleukin-6 (IL-6) and fractional iron absorption. A characteristic table was used to present extracted detail information of eligible studies. Following information were extracted: (1) the last name of the first author; (2) year of publication; (3) the study design (randomization, single- or double-blinded); (5) the population and health status of participants; (6) the duration and type of intervention in comparison and experiment groups; (7) number of participants in the intervention and comparison groups; and (8) outcomes. If outcomes measures were not reported as means and SDs, the following calculation processes were adopted to obtain the means and SDs: outcomes presented as the “median and range” were converted using VassarStats (Richard Lowry, New York, NY, USA) [29]; for studies not reporting the SD of mean difference, the following formula was used to estimate the SD of mean change between baseline and endpoint: SD change = square root [(SD_baseline_^2^ + SD_endpoint_^2^)/2].

### 2.2. Quality Assessment

RoB2 (Cochrane, London, United Kingdom) [30] was used to assess the risk of bias in included articles by two independent researchers. The following 5 domains were considered to assess the quality of clinical trials: (1) possibly bias from unjustified randomized process; (2) bias caused by deviation from intended interventions; (3) bias due to missing data; (4) bias from measurement of the outcome; (5) bias in selection of the reported result. The tool contains algorithms which calculate the risk-of-bias based on response to each question. The possible risk-of-bias judgments were as follows: (1) low risk-of-bias (represented by a green mark); (2) some concerns (represented by a yellow mark); and (3) high risk of bias (represented by a red mark). The assessment results were presented by Review Manager (RevMan, version 5.4; Cochrane, London, United Kingdom).

### 2.3. Statistical Analysis

Effect sizes were expressed as the weighted mean difference (WMD) and 95% confidence interval (CI) using random-effects model. Changes in the means and SDs of iron status parameters were considered as the representation of overall effects of iron supplement in intervention groups. The heterogeneity among the studies was assessed using the *I*^2^ statistic. Funnel’s plot and Egger test were used for the evaluation of publication bias. Sensitive analysis was used to judge the influence of individual study in pooled effect size by omitting one study. Forest plot was generated using RevMan (version: 5.4); funnel’s plot and sensitive analysis were performed in Stata/MP 16.0 (StataCorp, College Station, TX, USA).

## 3. Results

### 3.1. Study Characteristic

**Figure 1:**
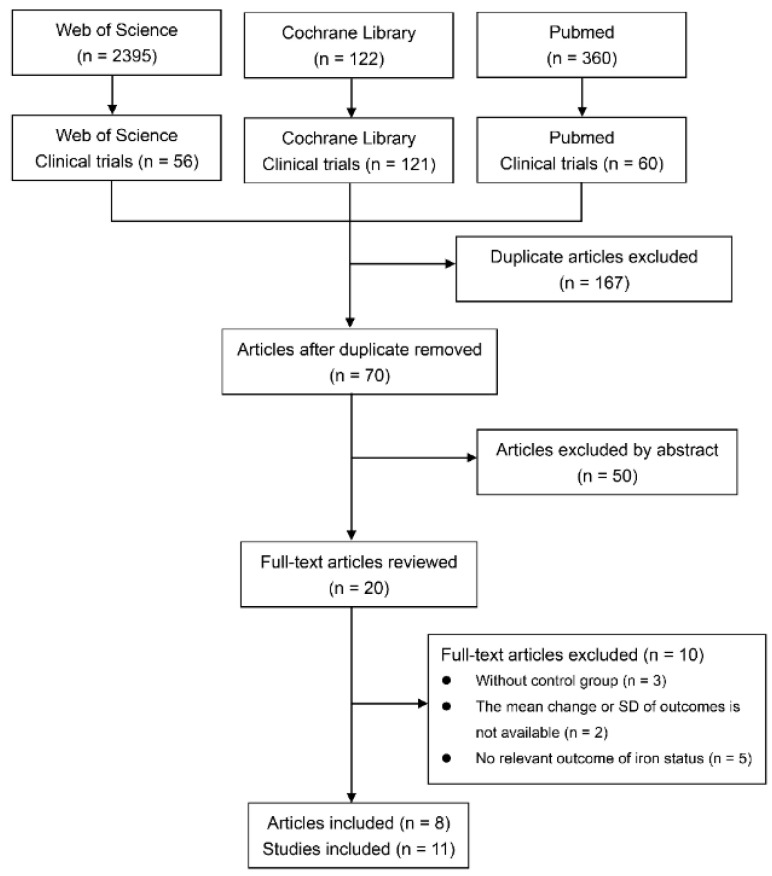
Study identification and selection flow diagram.

The literature search was conducted with key words mentioned in the method to identify studies comparing the effects of lactoferrin and ferrous sulfate on iron status or erythropoiesis. We initially identified 237 clinical trials articles, 60 from PubMed, 56 from Web of Science, and 121 from Cochrane Library. Seventy articles were reserved after removing duplication. Based on abstracts, 22 articles were reserved for full-text examination. Trials on pregnant and non-pregnant women reported in the same articles were regarded as separate studies. For trials with different intervention durations or doses in the same article, study with the longest intervention duration or the maximum dose were adopted. Finally, 9 randomized trials and 2 non-randomized trials from 8 articles were included for further analysis. Literature searching and selection process are presented in Figure 1. Of the 11 eligible studies, 8 studies were conducted on individuals with ID or IDA [31,32,33,34,35,36]; 6 studies were performed in pregnant women [31,32,33,34,35,36] and 4 studies in non-pregnant women [34,36,37]. There are 8 studies reporting iron status outcomes, including ferritin [33,34,35], serum iron [32,33,34,35,36] and hemoglobin concentration [31,32,33,34,35,36]; 3 studies measured the iron absorption [37,38]. A total of 680 participants were assigned to lactoferrin group and 582 participants were assigned to ferrous sulfate group. The characteristics of the included studies are presented in Table S1.

### 3.2. Effect of Lactoferrin and Ferrous Sulfate Supplementation on SI

**Figure 2:**
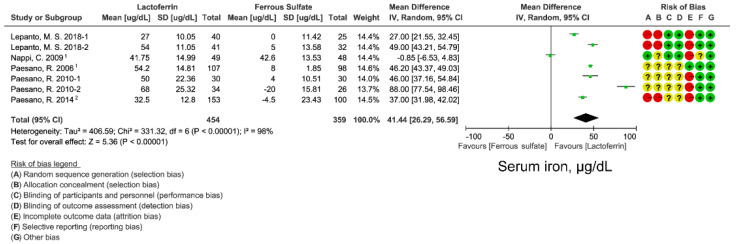
Effect of lactoferrin and ferrous sulfate on serum iron (SI). 1, converted from median and minimum-maximum range. 2. Mean and SD were estimated from histogram. Risk of bias legend: A = random sequence generation (selection bias); B = allocation concealment (selection bias); C = blinding of participants and personnel (performance bias); D = blinding of outcome assessment (detection bias); E = incomplete outcome data (attrition bias); F = selective reporting (reporting bias); G = other bias. Data expressed as weighted mean difference (95% CI). IV, inverse variance.

SI was used to assess the iron supplementation effect of lactoferrin and ferrous sulfate on circulating iron concentrations. Means and SD data were extracted from 8 studies. Random-effects model was used to evaluate the WMD of SI after lactoferrin and ferrous sulfate supplementation. Lactoferrin had a superior effect on SI compared with ferrous sulfate (WMD: 41.44 ug/dL; 95% CI: 26.29, 56.59; *I*^2^ = 98%; Figure 2). Although heterogeneity was observed across studies, no publication bias was found by funnel plot (Figure S1) and Egger’s test (*p* = 0.990; Table S2). Sensitive analysis also indicated that there was no single study affecting the total effect size (Figure S2).

### 3.3. Effect of Lactoferrin and Ferrous Sulfate Supplementation on SF

**Figure 3:**
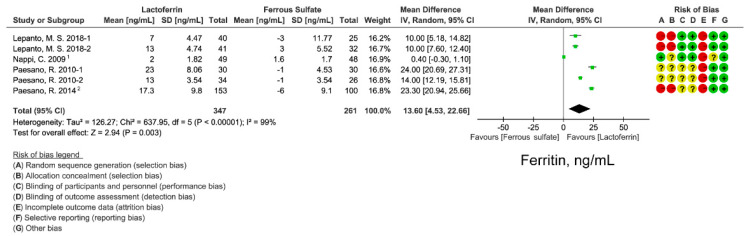
Effect of lactoferrin and ferrous sulfate on serum ferritin (SF) concentrations. 1. converted from median and minimum-maximum range. 2. Mean and SD were estimated from histogram. Risk of bias legend: A = random sequence generation (selection bias); B = allocation concealment (selection bias); C = blinding of participants and personnel (performance bias); D = blinding of outcome assessment (detection bias); E = incomplete outcome data (attrition bias); F = selective reporting (reporting bias); G = other bias. Data expressed as weighted mean difference (95% CI). IV, inverse variance.

We examined iron supplementation effect on iron storage level by analyzing serum ferritin from 6 studies. Lactoferrin still had a better effect on SF than ferrous sulfate (WMD: 13.6 ng/mL; 95% CI: 4.53, 22.66; *I*^2^ = 99%; Figure 3). Significant heterogeneity was observed across studies. Funnel plot (Figure S1) and Egger’s test (*p* = 0.036; Table S2) indicated that a small study effect may exist in the effect size. However, sensitivity analysis suggested that the effect size was robust (Figure S2).

### 3.4. Effect of Lactoferrin and Ferrous Sulfate Supplementation on Hemoglobin Concentration

**Figure 4:**
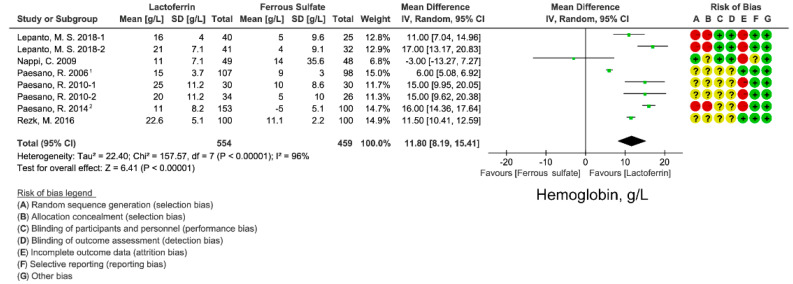
Effect of lactoferrin and ferrous sulfate on hemoglobin concentrations. 1, converted from median and minimum-maximum range. 2. Mean and SD were estimated from histogram. Risk of bias legend: A = random sequence generation (selection bias); B = allocation concealment (selection bias); C = blinding of participants and personnel (performance bias); D = blinding of outcome assessment (detection bias); E = incomplete outcome data (attrition bias); F = selective reporting (reporting bias); G = other bias. Data expressed as weighted mean difference (95% CI). IV, inverse variance.

Most participants included in this study are pregnant women who are at high risk for IDA. Hemoglobin is the major blood determinant in the clinical diagnosis of IDA. We extracted and analyzed hemoglobin concentration data from 9 studies. Analysis results using random-effects model favored oral lactoferrin intervention over ferrous sulfate with a WMD of 11.80 g/L (95% CI: 8.19, 15.41; *I*^2^ = 96%; Figure 4), which indicates the better erythropoiesis improving effect of oral lactoferrin. There was no publication bias of hemoglobin concentration in the included studies, based on funnel plot (Figure S1) and Egger’s test (*p* = 0.376; Table S2). Sensitivity analysis indicated that no single study affected the total effect size of hemoglobin concentration (Figure S2).

### 3.5. Effect of Lactoferrin and Ferrous Sulfate Supplementation on FIA

**Figure 5:**
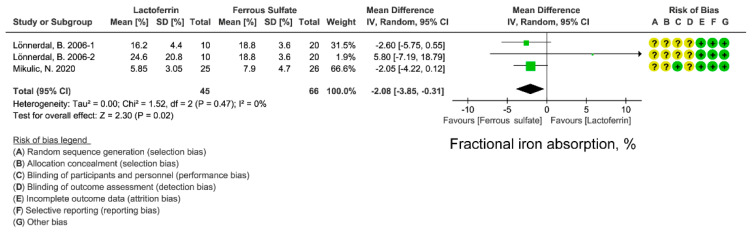
Effect of lactoferrin and ferrous sulfate on fractional iron absorption (FIA). Risk of bias legend: A = random sequence generation (selection bias); B = allocation concealment (selection bias); C = blinding of participants and personnel (performance bias); D = blinding of outcome assessment (detection bias); E = incomplete outcome data (attrition bias); F = selective reporting (reporting bias); G = other bias. Data expressed as weighted mean difference (95% CI). IV, inverse variance.

We next compared the effects of lactoferrin and iron ferrous on iron absorption. FIA can better reflect iron absorption from intestine. FIA is calculated from iron-isotopic ratios, iron circulating in the body, and the total amounts of isotope-labeled iron administrated in the participant [37,38]. Although lactoferrin displayed a superior effect on circulating iron, iron storage level (Figure 2, Figure 3 and Figure 4), lactoferrin had an unexpectedly inferior effect on FIA when compared with ferrous sulfate (WMD: −2.08%; 95% CI: −3.85, 0.31; *I*^2^ = 0%; Figure 5). There is low heterogeneity in included studies and no publication bias was found in FIA (Figures S1 and S2; Table S2).

### 3.6. Effect of Lactoferrin and Ferrous Sulfate Supplementation on Inflammatory Cytokine IL-6

**Figure 6:**
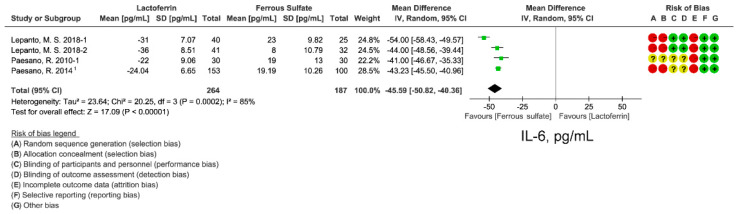
Effect of lactoferrin and ferrous sulfate on serum IL-6 levels. 1. Mean and SD were estimated from histogram. Risk of bias legend: A = random sequence generation (selection bias); B = allocation concealment (selection bias); C = blinding of participants and personnel (performance bias); D = blinding of outcome assessment (detection bias); E = incomplete outcome data (attrition bias); F = selective reporting (reporting bias); G = other bias. Data expressed as weighted mean difference (95% CI). IV, inverse variance.

Lactoferrin has immunomodulatory activity [39]. Inflammatory cytokines IL-6 levels were extracted from 4 studies to evaluate inflammatory status after lactoferrin or ferrous sulfate intervention. Lactoferrin supplementation group had a lower IL-6 levels (WMD: −45.59 pg/mL; 95% CI: −50.82, −40.36; *I*^2^ = 85%; Figure 6) compared with ferrous sulfate (Figure 6). Funnel plot (Figure S1) and Egger’s test (*p* = 0.345; Table S2) were performed to evaluate publication bias and no bias was observed. Sensitive analysis also indicated a reliable effect of lactoferrin on IL-6 (Figure S2).

### 3.7. Subgroup Analyses of Ferrous Sulfate Supplementation on Iron Status

We further identified studies according to 2 factors which might affect outcomes, including participants’ populations (pregnant women or non-pregnant women) and health status (the presence of ID/IDA or hereditary thrombophilia). No significant difference was observed in the WMDs of SI, ferritin, hemoglobin and IL-6 levels among subgroups. WMD, heterogeneity within groups and meta-regression results were presented in Table S3.

## 4. Discussion

Lactoferrin is a multifunctional iron-binding protein possessing anti-inflammatory and anti-microbial effects. The major advantages of lactoferrin compared with traditional iron supplement ferrous sulfate are well gastrointestinal tolerance and fewer side effects caused by the intake of high-dose iron. In this meta-analysis, we compared the iron- and erythropoiesis-improving effects between lactoferrin and ferrous sulfate based on 11 intervention studies. Lactoferrin supplementation displayed better improvement for blood iron parameters including serum iron (Figure 3) and ferritin (Figure 2) when compared with ferrous sulfate. Moreover, a superior erythropoiesis-improving effect was observed in individuals receiving lactoferrin (Figure 4). Participants receiving lactoferrin had reduced IL-6 levels compared with those receiving ferrous sulfate, indicating lactoferrin mitigated inflammation.

Contradictorily, lactoferrin has less FIA than ferrous sulfate based on 3 studies (Figure 5) [37,38], suggesting that the iron-improving effect of lactoferrin is not completely brought by the iron within lactoferrin. The lower FIA of lactoferrin is probably due to its iron-binding activity, which sequesters iron rather than promotes iron absorption in the intestine [38]. This hypothesis can be supported by the phenotypes of lactoferrin knockout mice, in which increased serum transferrin saturation and liver iron stores were observed [40]. However, there is an inconsistent explanation about the effect of iron-binding capacity of lactoferrin on iron absorption. It regarded that lactoferrin may sequester dietary iron from binding to iron-chelating compounds in foods (such as polyphenols and phytic acid), which promotes extra iron absorption in foods. This hypothesis can be supported by the results from isotope-labeled iron absorption trial using apo-lactoferrin [38]. Higher FIA was observed in individuals receiving apo-lactoferrin than those who received holo-lactoferrin.

**Figure 7:**
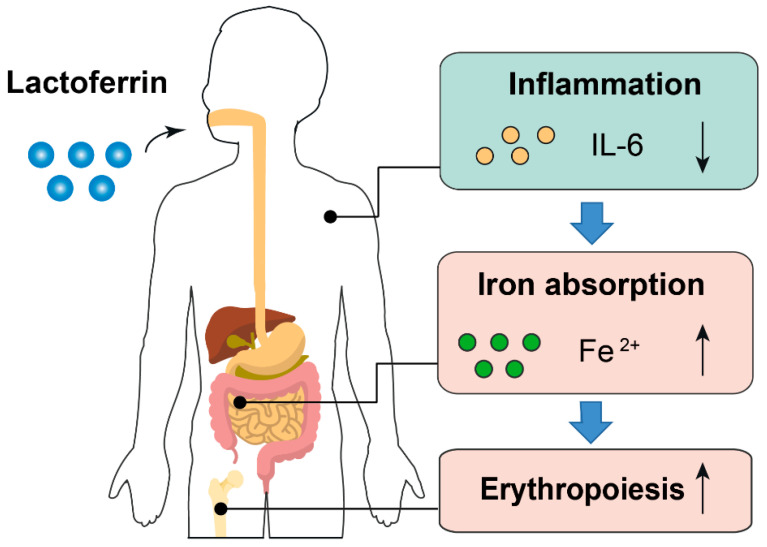
Proposed model describing the iron- and erythropoiesis-improving effects of lactoferrin. Part of the elements in this figure are using resources from Freepik [47] and Servier Medical Art [48] under a Creative Commons Attribution license.

Based on the results of this study, we proposed that the anti-inflammatory effect of lactoferrin can partially explain why it had a better iron-improving effect even with lower FIA than ferrous sulfate. First, higher inflammatory cytokines will prevent dietary iron absorption and iron mobilization from reticuloendothelial system to meet the need of erythropoiesis because of increased hepcidin synthesis [41,42]. Second, amelioration of inflammation can improve erythropoiesis. Inflammatory cytokines such as IL-6 is a repressive factor for erythropoiesis [43] (Figure 7). Notably, the iron-binding capacity also confers lactoferrin the property of limiting the growth of gut pathogens, which requires iron to proliferate. Simultaneously, lactoferrin can also enhance the growth of gut-beneficial microorganisms such as *Bifidobacterium* and *Lactobacillus* [44]. These probiotics have been proven to reduce the adverse effects associated with iron supplementation [45,46].

This meta-analysis has several limitations. First, only 8 articles and 1262 participants were included into analysis, which may affect the reliability of the results. For example, the results of IL-6 and FIA were based on data extracted from 3 and 2 articles [33,34,36,37,38]. Moreover, the participants number in these studies is relatively small. Therefore, the comparative effects of lactoferrin on IL-6 and FIA should be cautiously interpreted. Second, 3 included studies did not have randomization [33,36] or blinding [31,32,33,34,36,37]. These risks of bias may downgrade the evidence level of corresponding outcomes.

Whether lactoferrin promotes iron status by mitigating inflammation still needs validating in future studies. Inflammatory cytokines such as IL-6, CRP and TNF-α should be detected in further trials. With respect to the lower fractional iron absorption of lactoferrin compared with ferrous sulfate (Figure 5), more iron absorption investigations on lactoferrin are required. A critical serum parameter needs detection in future trials is hepcidin, especially considering the essential function of hepcidin in iron metabolism [49]. Currently, the effect of lactoferrin on hepcidin remains unclear, and the measurement of serum hepcidin will help to explain the modulatory mechanism of lactoferrin on iron absorption. Moreover, the absorption efficiency difference between apo-lactoferrin and holo-lactoferrin may be another aspect for the further exploration of how lactoferrin modulates iron absorption. Iron absorption efficiency can be detected in animals or human volunteers receiving lactoferrin with different iron saturation levels. It may help to understand the interaction between apo-lactoferrin and dietary free iron in the gut, as well as their effects on intestinal iron absorption.

In conclusion, this study provides evidence to support lactoferrin as a superior supplement to ferrous sulfate to improve serum iron, ferritin and hemoglobin levels. Lactoferrin-bound iron is not an iron supplement per se, but immune modulator affecting iron homeostasis via lactoferrin-dependent signal transduction mechanism [9]. The anti-inflammation effect of lactoferrin may be the potential mechanism to explain its efficacy on iron status and erythropoiesis. Further intervention and mechanistic studies are warranted to explore the functions of lactoferrin in the regulation of iron absorption. 

## Data Availability

Not applicable.

## Supplementary Materials

The following supporting information can be downloaded at: https://www.mdpi.com/article/10.3390/nu14030543/s1, Table S1: Characteristics of the publications included in the meta-analysis; Table S2: Egger’s linear regression tests of the publications included in the meta-analysis; Table S3: Subgroup analyses of iron parameters and IL-6; Figure S1: Funnel plots for analyzing publication bias; Figure S2: Sensitivity analysis for the included studies.
