# Supplementary material for: Comparative Effects between Oral Lactoferrin and Ferrous Sulfate Supplementation on Iron-Deficiency Anemia: A Comprehensive Review and Meta-Analysis of Clinical Trials
Source: Nutrients. 2022 Jan 27;14(3):543. doi: 10.3390/nu14030543 (PMC8838920; doi:10.3390/nu14030543)
Supplement: Supplementary file 1 [file nutrients-14-00543-s001.zip › nutrients-1543765-supplementary.pdf]

Comparative effects between oral lactoferrin and ferrous sulfate supplementation on iron-deficiency anemia: a comprehensive review and meta-analysis of clinical trials

Table S1. Characteristics of the publications included in the meta-analysis.

Table S2. Egger's linear regression tests of the publications included in the meta-analysis.

Table S3. Subgroup analyses of iron parameters and IL-6.

Figure S1. Funnel plots for analyzing publication bias.

Figure S2. Sensitivity analysis for the included studies.

Supplementary Table S1. Characteristics of the publications included in the meta-analysis.

Article (first author, year)	Study design	Populations	Health Status	Duration	Intervention	N (Lf, Fe)	Extracted outcome(s)
Rezk, 2016	R	pregnant women	IDA	8W	lactoferrin; ferrous sulfate	200 (100,100)	Hb
Paesano, 2006	R	pregnant women	ID or IDA	30D	lactoferrin; ferrous sulfate	205 (107,98)	Hb, TSI
Paesano, 2010-1	R	pregnant women	ID or IDA	30D	lactoferrin; ferrous sulfate	60 (30,30)	Hb, TSI, SF, IL-6
Paesano, 2010-2	R	non-pregnant women	ID or IDA	90D	lactoferrin; ferrous sulfate	60 (34,26)	Hb, SI, SF
Nappi, 2009	R, DB	pregnant women	IDA	30D	lactoferrin; ferrous sulfate	97 (49,48)	Hb, SI, SF
Paesano, 2014	-	pregnant women	ID or IDA, hereditary thrombophilia	Intervened until delivery	lactoferrin; ferrous sulfate	253 (153,100)	Hb, SI, SF, IL-6
Lepanto, 2018	-	pregnant	hereditary thrombophilia	30D	lactoferrin; ferrous sulfate	65 (40,25)	Hb, SI, SF, IL-6
Lepanto, 2018	-	child-bearing women	hereditary thrombophilia	30D	lactoferrin; ferrous sulfate	73 (41,32)	Hb, SI, SF, IL-6
Mikulic, 2020	R, SB	infants	healthy	28D	holo-lactoferrin; ferrous sulfate	51 (25,26)	FIA

Lönnerdal, 2006-1	R	non-pregnant women	healthy, slightly anemic or ID	28D	lactoferrin; ferrous sulfate	30 (10,20)	FIA
Lönnerdal, 2006-2	R	non-pregnant women	healthy, slightly anemic or ID	28D	heat treated lactoferrin; ferrous sulfate	30 (10,20)	FIA

Lf, Lactoferrin group; CVD; D, day; DB, double-blinded; SB, single-blind; SF, Serum ferritin; Hb, Hemoglobin; Fe, ferrous sulfate group; W, week; FIA, Fractional iron absorption; IL-6, Interleukin-6; N, Number of people involved; ID, Iron deficiency; R, randomized; IDA, iron-deficiency anemia; SI, Serum iron.

Supplementary Table S2. Egger's tests of the publications included in the meta-analysis.

Indicators	*P* value of Egger's tests
Serum iron	0.990
Serum ferritin	0.036
Hemoglobin concentration	0.376
FIA	0.345
IL-6	0.689

FIA, fractional iron absorption; IL-6, Interleukin-6.

Supplementary Table S3. Subgroup analyses of iron parameters and IL-6.

	N	WMD (95% CI)	I^2 (%)	*P* value[1]	*P* value[2]
Serum ferritin, ug/dL					
Populations					
pregnant women	4	14.41 (-0.45, 29.28)	99	<0.0001	0.796
non-pregnant women	2	12.08 (8.16, 16.00)	85	0.009	
Health Status					
ID or IDA	3	12.72 (-0.32, 25.77)	97	<0.0001	0.836
hereditary thrombophilia	3	14.52 (4.73, 24.31)	99	<0.0001	
Serum iron, ng/mL					
Populations					
pregnant women	5	31.03 (14.22, 47.85)	98	<0.0001	0.094
non-pregnant women	2	68.25 (30.03, 106.46)	98	<0.0001	
Health Status					
ID or IDA	4	44.61 (14.53, 74.69)	99	<0.0001	0.766
hereditary thrombophilia	3	37.63 (25.65, 49.61)	93	<0.0001	
Hemoglobin, g/dL					
Populations					
pregnant women	6	10.45 (6.36, 14.54)	96	<0.0001	0.260
non-pregnant women	2	16.33 (13.21, 19.45)	0	0.55	
Health Status					
ID or IDA	5	9.90 (5.79, 14.02)	95	<0.0001	0.253
hereditary thrombophilia	3	14.91 (11.85, 17.97)	66	0.05	
IL-6, pg/mL					
Populations					
pregnant women	3	-47.63 (-60.36, -34.89)	92	0.0004	0.817
non-pregnant women	1	-44.00 (-48.56, -39.44)	—	—	
Health Status					
ID or IDA	1	-41.00 (-46.67, -35.33)	—	—	0.497
hereditary thrombophilia	3	-46.93 (-53.39, -40.47)	89	0.0001	

ID, iron deficiency; IDA, iron deficiency amenia; IL-6, Interleukin-6.

[1] *P* values for heterogeneity within each subgroup.

[2] *P* values for heterogeneity between groups from metaregression.

—, data not available; N, the number of included studies; WMD, weighted mean difference.

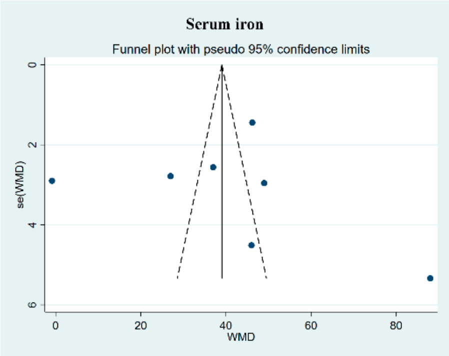

(a)

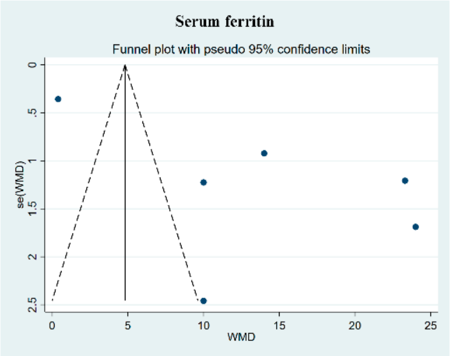

(b)

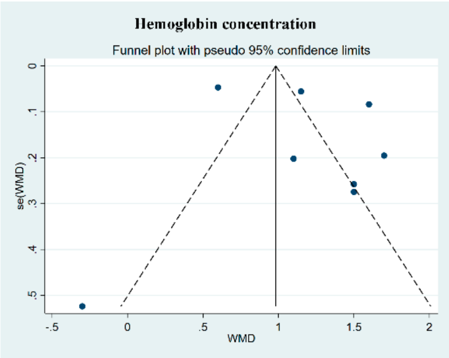

(c)

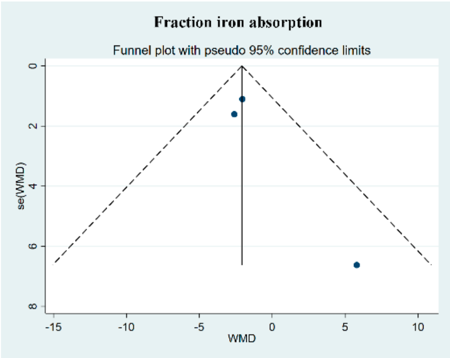

(d)

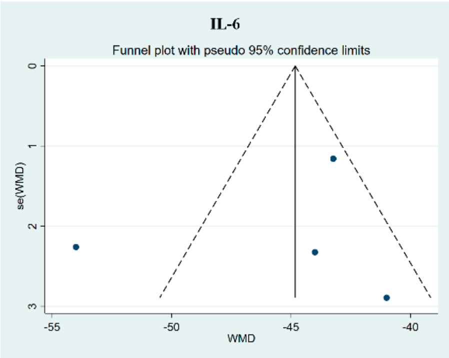

(e)

Supplementary Figure S1. Funnel plots for analyzing publication bias. Funnel plots with pseudo 95% confidence limits were generated in order to identify possible publication bias of the included studies. The mean differences are plotted against the standard error for the indicated indicators. Each symbol represents a single study. IL-6, Interleukin-6.

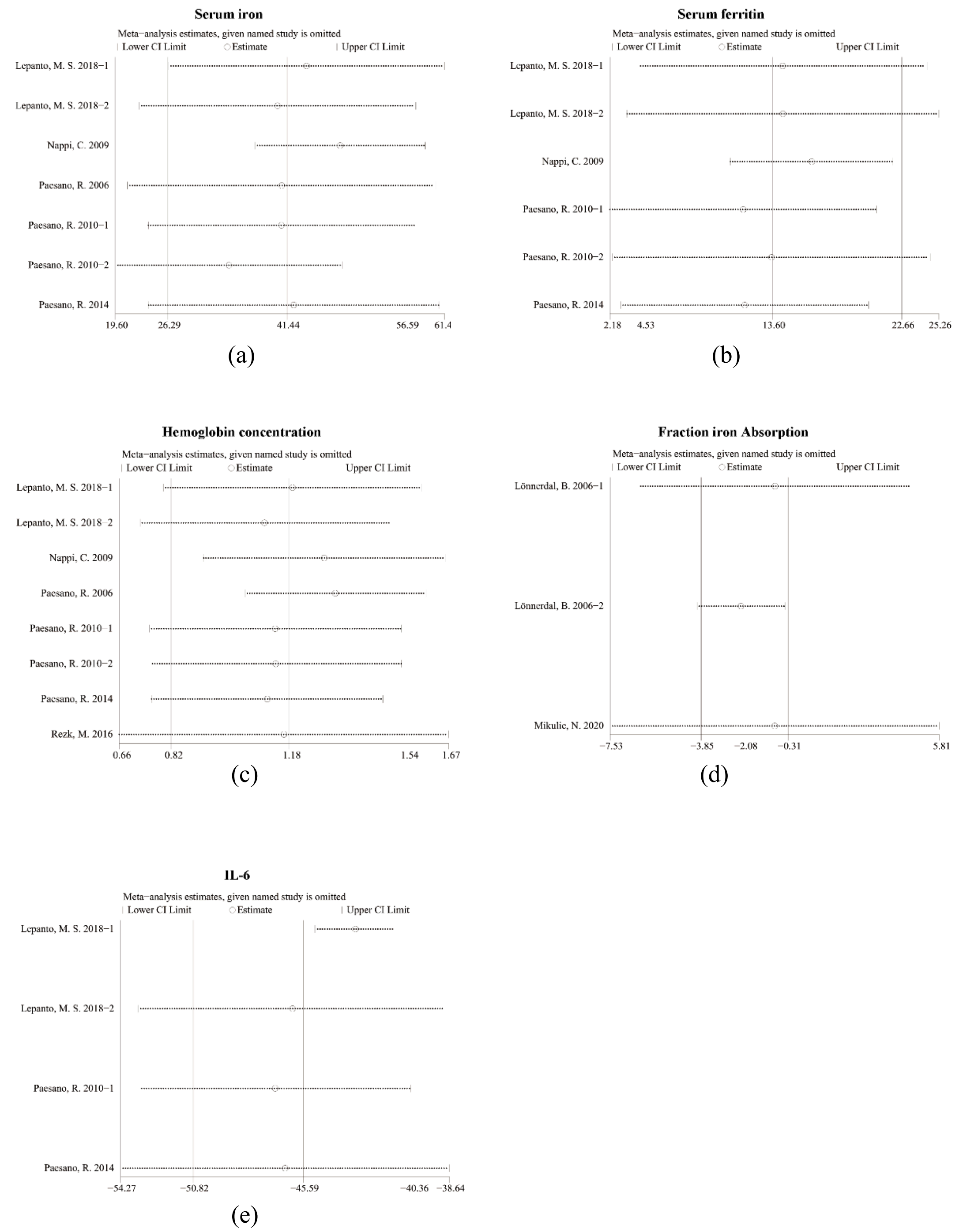

Supplementary Figure S2. Sensitivity analysis for the included studies. The results of a sensitivity test are shown for the indicated indicators. In each panel, each indicated study was omitted from the pooled analysis, and the effect on the total results was determined. Each circle and corresponding vertical tick represent the effect size and 95% CI after the corresponding study was omitted. For comparison, the three vertical lines indicate the positions of the effect size and the upper and lower limits of the 95% CI for the pooled results. IL-6, Interleukin-6.
